# Migraine induction with calcitonin gene-related peptide in patients from erenumab trials

**DOI:** 10.1186/s10194-018-0927-2

**Published:** 2018-11-08

**Authors:** Casper Emil Christensen, Samaira Younis, Marie Deen, Sabrina Khan, Hashmat Ghanizada, Messoud Ashina

**Affiliations:** Danish Headache Center and Department of Neurology, Rigshospitalet Glostrup, Faculty of Health and Medical Sciences, University of Copenhagen, Copenhagen, Denmark

**Keywords:** Headache, CGRP, Biomarker, Monoclonal antibody

## Abstract

**Background:**

Migraine prevention with erenumab and migraine induction by calcitonin gene-related peptide (CGRP) both carry notable individual variance. We wanted to explore a possible association between individual efficacy of anti-CGRP treatment and susceptibility to migraine induction by CGRP.

**Methods:**

Thirteen migraine patients, previously enrolled in erenumab anti-CGRP receptor monoclonal antibody trials, received CGRP in a double-blind, placebo-controlled, randomized cross-over design to investigate their susceptibility to migraine induction. A standardized questionnaire was used to assess the efficacy of previous antibody treatment. The patients were stratified into groups of high responders and poor responders. Primary outcomes were incidence of migraine-like attacks and area under the curve of headache intensity after infusion of CGRP and placebo. All interviews and experiments were performed in laboratories at the Danish Headache Center, Copenhagen, Denmark.

**Results:**

Ten high responders and three poor responders were included. CGRP induced migraine-like attacks in ten (77%) patients, whereof two were poor responders, compared to none after placebo (*p* = 0.002). The area under the curve for headache intensity was greater after CGRP, compared to placebo, at 0–90 min (*p* = 0.009), and 2–12 h (*p* = 0.014). The median peak headache intensity score was 5 (5–9) after CGRP, compared to 2 (0–4) after placebo (*p* = 0.004).

**Conclusions:**

Patients with an excellent effect of erenumab are highly susceptible to CGRP provocation. If an association is evident, CGRP provocation could prove a biomarker for predicting antibody treatment efficacy.

**Trial registration:**

Retrospectively registered at clinicaltrials.gov with identifier: NCT03481400.

## Background

**Fig. 1:**
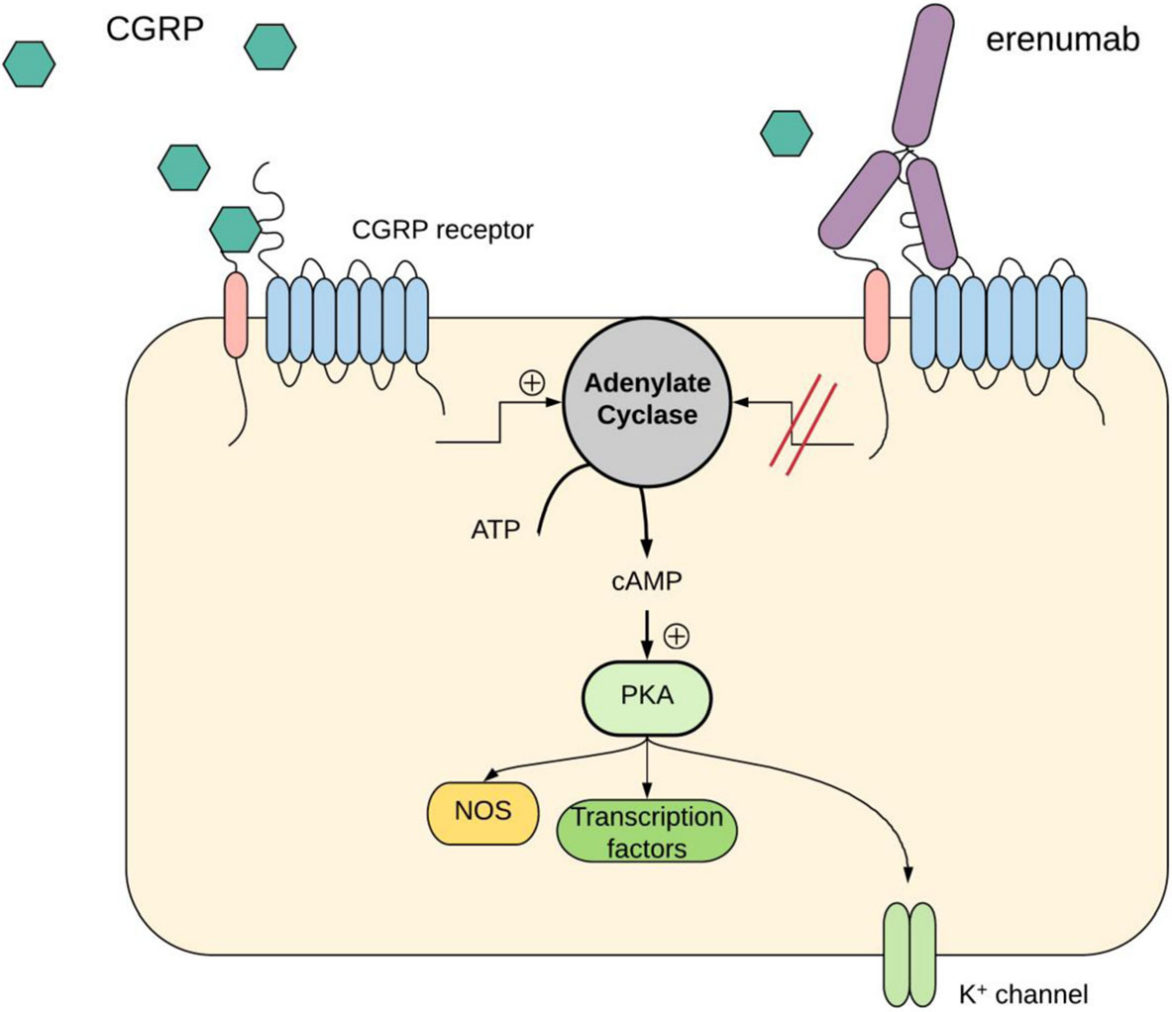
Intracellular signaling pathways of calcitonin gene-related peptide receptor activation. One effect of CGRP receptor activation is adenylate cyclase-mediated cyclic adenosine monophosphate (cAMP) elevation, which leads to protein kinase A (PKA) activation, and activation of multiple targets depending on cell type. Nitric oxide synthesis may be the result of nitric oxide synthase (NOS) phosphorylation, gene transcription changes may be a result of cAMP response element binding protein (CREB) activation, and relaxation of vascular smooth muscle cells is partly a result of ATP-sensitive potassium channels (K+ channels) activation

Clinicians treating migraine have, until now, been limited to preventive drugs that were initially developed for cardiovascular, psychiatric or neurological diseases other than migraine. [1] Four anti calcitonin gene-related peptide (anti-CGRP) monoclonal antibodies (mAbs) are in late-phase development as the first class of preventive therapeutics targeting migraine-specific mechanisms. [2] Three mAbs (fremanezumab, eptinezumab and galcanezumab) are ligand specific, and bind to CGRP, while one (erenumab) binds to the receptor complex (Fig. 1). [3–6] Overall efficacy and tolerability between the four antibodies are quite similar, but individual efficacy is widespread. While some patients report excellent efficacy, 35% report less than 50% reduction in monthly migraine days when treated with erenumab. [7] The question is whether we can identify which patients to treat with the new therapeutics by predicting efficacy response and thereby introduce personalized treatment schemes.

Calcitonin gene-related peptide induces migraine-like attacks in an average of 62% of migraine patients across placebo-controlled and open-label provocation studies. [8–11] Individual differences in mAb efficacy and migraine induction suggest that CGRP involvement in migraine varies between patients, and susceptibility to provocation could be a possible biomarker for anti-CGRP treatment efficacy.

We sought to investigate a possible association between anti-CGRP treatment efficacy and susceptibility to CGRP-induced migraine-like attacks. Our hypotheses were that CGRP would conduce to a small migraine-like attack rate in a group of patients with little to no effect of erenumab and a large attack rate in a group who experienced an excellent effect of erenumab.

## Methods

### Recruitment process

Patients, who had participated in the episodic and chronic erenumab trials (ClincalTrials.gov IDs: NCT02483585 and NCT02066415), were recruited from the Danish Headache Center. These patients were contacted and enrolled after completing their participation in the mAb trial. Patients, who were likely eligible for participation in up-coming anti-CGRP mAbs clinical trials, were recruited from the Danish Headache Center as well. The patients were enrolled from July 25 2016 to June 21 2017. Inclusion criteria: migraine with and/or without aura according to the International Classification of Headache Disorders (ICHD-3 beta) [12], age 18 to 65 years, and previous/probable participation in an anti-CGRP mAb trial. Exclusion criteria: use of pharmacological agents (except contraceptives and preventive migraine medication), cardiovascular disease and other serious somatic or psychiatric disorders.

### Study design

**Fig. 2:**
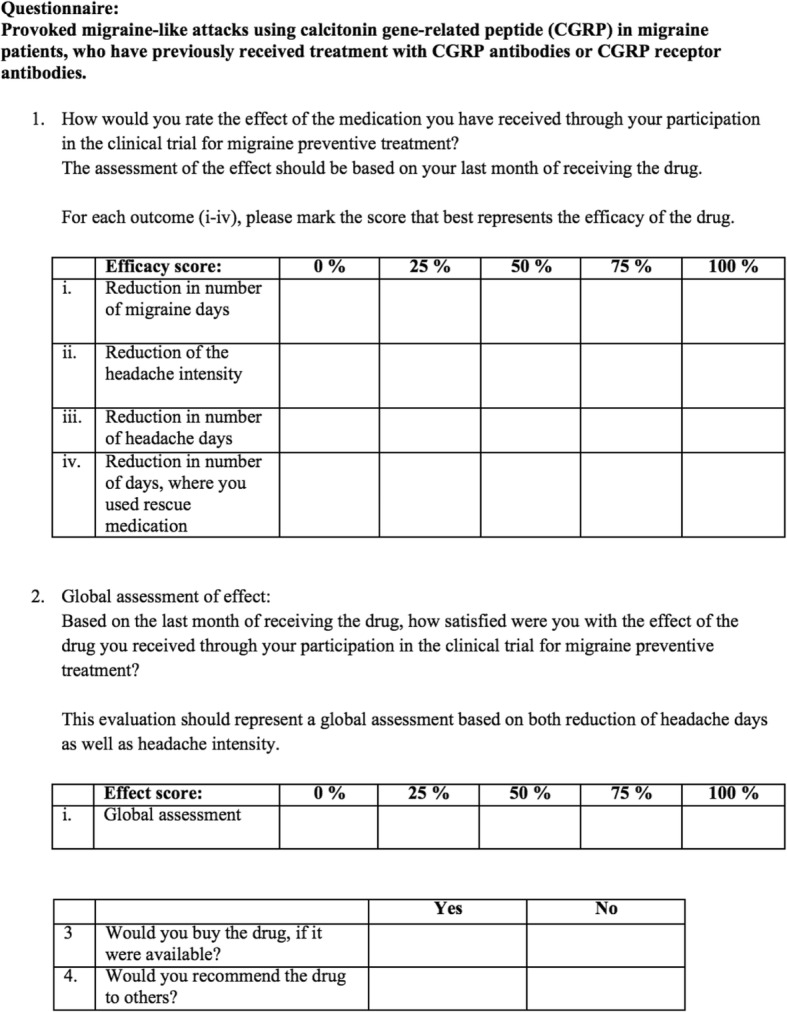
Questionnaire used for monoclonal antibody response stratification. Patients who reported excellent effect of treatment (efficacy score ≥ 50%) in at least two of the four outcome variables (i-iv) were defined as high responders. The remaining patients were defined as poor responders

Response to anti-CGRP mAb treatment was evaluated using a standardized questionnaire (Fig. 2). Patients rated treatment efficacy for reduction in: migraine days, headache days, days using rescue medication, and headache intensity. Treatment efficacy was assessed based on the patients’ last month of receiving mAbs. Patients, who reported an excellent effect of treatment (efficacy score ≥ 50%) in at least two of the four outcome variables, were defined as high responders. The remaining patients were defined as poor responders.

Patients received 1.5 μg/min human α-CGRP (PolyPeptide, Strasbourg, France) and placebo isotonic saline as infusions over 20 min on two separate study days in a double-blind, placebo-controlled, randomized, cross-over design.

### Experimental protocol

Patients reported to the clinic headache-free for at least 48 h. Coffee, tea, cocoa, cola, tobacco, and alcohol were not allowed for 12 h before study start. Patients were instructed to fast for four hours before study start. Fertile female participants underwent a pregnancy test upon arrival at the hospital.

Patients underwent a medical examination on the first study day. A venous catheter was inserted into a cubital vein, followed by rest in supine position for 30 min, before initiating the infusion. Intensity and characteristics of headache, heart rate (HR), blood pressure, and adverse events were registered every 10 min from 10 min before to 90 min after infusion.

### Headache intensity and characteristics

Headache intensity was rated based on a 0 to 10 numeric rating scale (NRS) where ‘0’ denoted no headache, and ‘10’ the worst possible headache.

Headache characteristics were recorded using a standardized questionnaire including headache intensity, location, quality, aggravation by physical activity, and accompanying symptoms.

Upon discharge from the hospital, patients were instructed to self-report headache intensity and characteristics in a standardized headache diary hourly from 2 to 12 h after infusion start. Patients were allowed to use their usual migraine medication after discharge.

### Migraine-like attack criteria

Pharmacologically-induced migraine attacks are not spontaneous attacks, and cannot fulfill the ICHD-3 beta criteria. [12] Therefore, modified criteria for experimentally-induced attacks were developed based on the following considerations. [13, 14] Firstly, the majority of patients report that the induced attacks mimic their spontaneous attacks. [10, 15] Secondly, spontaneous migraine attacks mostly develop in a matter of hours, and in the beginning of the attack phenomenologically fulfill the criteria for tension-type headache. Only hereafter, the headache worsens, becomes unilateral and presents the associated symptoms required for a migraine diagnosis. Finally, most patients can predict an impending migraine attack in the early attack stage and cannot be denied treatment in an experimental setting. Thus, induced attacks are frequently treated before all migraine criteria are fulfilled. Accordingly, we used the following two criteria to define a pharmacologically-induced migraine-like attack [14]:

The headache fulfills criteria C and D of the ICHD-3 beta [12].

- C: Headache has at least two of the following characteristics: Unilateral location, pulsating quality, moderate to severe intensity, or aggravation by physical activity.
- D: At least one of the following accompanying symptoms: Nausea and/or vomiting, or photophobia and phonophobia.

*or*

Headache described as mimicking the patient’s spontaneous attack and treated with acute migraine rescue medication.

### Statistical analysis

Headache intensity scores are presented as median (range). Heart rate and mean arterial pressure (MAP) are presented as mean ± standard deviation under the assumption that they adhere to a normal distribution. Primary endpoints were incidence of migraine-like attacks from 0 to 12 h after infusion and area under the curve (AUC), using the trapezoidal rule [16], for headache intensity score at 0 to 90 min and 90 min to 12 h on the CGRP day, as compared to the placebo day for all patients. McNemar’s test and Wilcoxon signed-rank test were used as appropriate. Secondary endpoints were HR and MAP, which were compared between the two study days using paired t-tests. Peak headache intensity score and time to peak headache were compared between the study days using Wilcoxon signed-rank test. Adverse events are reported as incidences on the CGRP and placebo day and compared between days using McNemar’s test in explorative analysis. Predictive values, sensitivity and specificity were also calculated as post hoc analyses.

Separate meaningful inference statistics within each mAb response groups could not be performed due to small subgroup sample sizes. Data from patients without previous experience from the erenumab trials were excluded from the final analyses as this recruitment was limited by the competitive enrollment strategies of the anti-CGRP mAbs clinical trials. No statistical power calculation was conducted prior to the study as the sample size was based on the available data. R (Version 3.4.2) was used to conduct the statistical analyses. *P* values are reported as two-tailed with a 5% level of significance.

## Results

### Participants

**Fig. 3:**
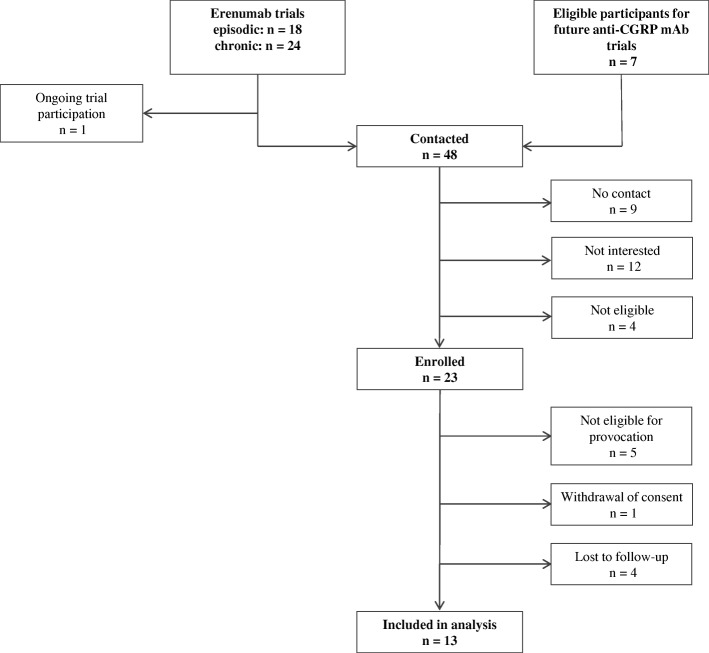
Inclusion flowchart. Twenty-three patients were enrolled in the study. Ten of these were excluded subsequently. One patient was excluded due to a cardiac conduction disease and one due to diabetes mellitus (well-regulated), according to the conventional CGRP provocation protocol. Three patients were excluded from analysis as they did not participate in the erenumab trials. One patient withdrew consent before the experiments. Four patients were lost to follow-up and one of these had participated in the first study day. Data from these days were excluded from analyses. Of the ten patients, who were excluded, seven had received erenumab and six of these were high responders. Response status was not obtained from the last of the seven subjects

Thirteen patients (12 women) completed the study (Fig. 3). Seven were enrolled from the episodic migraine erenumab trial (ClincalTrials.gov ID: NCT02483585), and six were enrolled from the chronic migraine erenumab trial (ClincalTrials.gov ID: NCT02066415). All 13 patients were enrolled after completing the safety follow-up visit 12 weeks after the last dose of erenumab. Mean age was 39 years (standard deviation ±11 and range 22 to 53).

### Clinical characteristics, migraine incidence and intensity

**Fig. 4:**
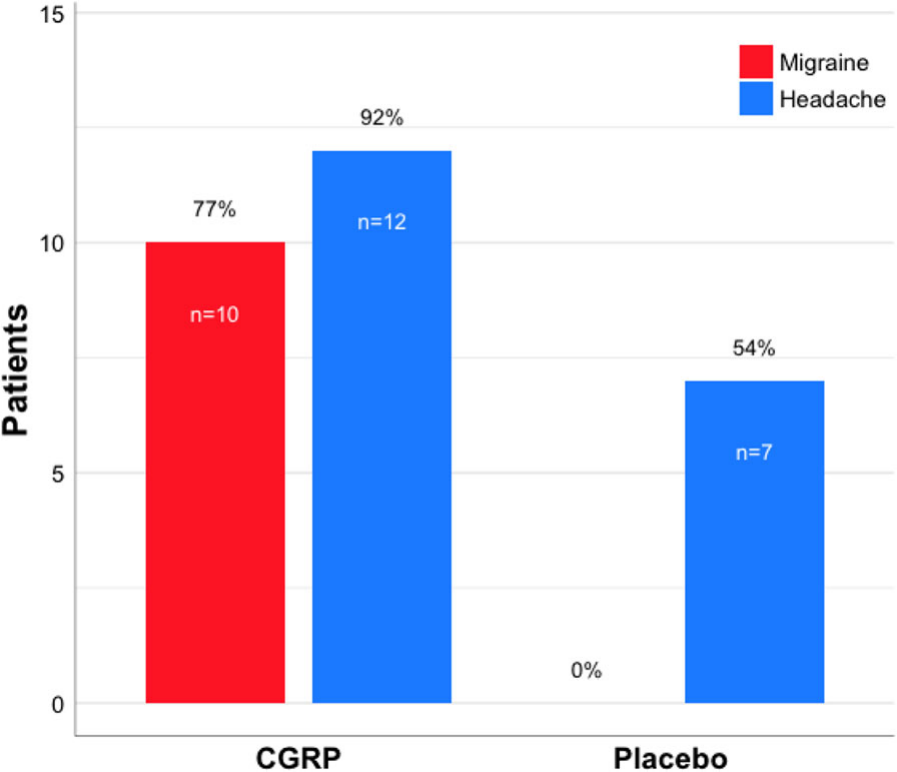
Proportion of patients who developed migraine-like attacks and headache after CGRP and placebo. More patients developed migraine-like attacks after CGRP (*n* = 10), compared to placebo (*n* = 0) (*p* = 0.002)

Headache characteristics and accompanying symptoms are presented in Table 1. Ten of 13 patients (77%) developed migraine-like attacks after CGRP, compared to none after placebo (*p* = 0.002) (Fig. 4). Two of the 10 patients, who experienced migraine-like attacks, reported poor response to treatment (patients 5 and 12).

**Table 1:** Clinical characteristics of headache and associated symptoms after CGRP and placebo

Patient	Efficacy score (%)	Day	Time to peak headache (duration)	Headache characteristics	Associated symptoms	Mimics usual migraine	Migraine-like attack (onset)	Treatment (time)/efficacy
1 CM	50/75/0/25	CGRP Placebo^a^ Spon	3 h (4 h) 80 min (NA)	Bilat/10/Throb+Pres/+ Bilat/6/Pres/M Bilat/Throb/+	+/+/+ +/+/− +/+/+	Yes No	Yes (20min) No	Sumatriptan 100 mg (6 h) / No NR
2 EM	100/100/ 100/100	CGRP Placebo Spon	2 h (NA)^b^ None	Bilat/10/Pres/+	−/+/−	Yes	Yes (20 min)	Sumatriptan 50 mg (2 h) / Yes
				Right/Throb/+	+/+/−			
3 EM	100/0/0/100	CGRP Placebo Spon	3 h (1 h) 30 min (1h)	Bilat/7/Throb/+ Bilat/3/Pres/M Left/ Throb/+	+/+/+ −/+/− +/+/+	Yes No	Yes (70 min) No	Sumatriptan 50 mg (3 h) / Yes, Treo (9 h) / NR None
4 EM	100/50/0/100	CGRP^c^ Placebo Spon	6 h (1 h) 10 h (NA)	Bilat/7/Throb+Pres/NR Left/5/Throb/+ Left/Throb/+	+/+/+ −/−/+ +/+/+	NR Yes	Yes (5 h) No (NA)^e^	2 x KP (5 h) / No, Riza 10 mg (6 h) / NR^d^, KP (10 h) / Yes Riza + 2 x KP (10 h) / NR^f^
5 EM	0/25/0/0	CGRP Placebo Spon	3 h (1 h) None	Bilat/5/Pres/+ Bilat/Throb/+	−/+/+ −/+/+	Yes	Yes (20 min)	Sumatriptan 100 mg (3 h) / Yes
6 EM	75/50/50/75	CGRP Placebo Spon	3 h (2 h) 9 h (2 h)	Right/4/Pres/− Right/3/NR/- Unilat^g^/Throb/+	−/+/− −/+/− +/+/+	Yes Yes	Yes (2 h) No (NA)	Zolmitriptan 2.5 mg (3 h) / Yes, 2 x Treo (6 h) / Yes Treo (9 h) / NR
7 EM	75/75/0/75	CGRP Placebo Spon	1 h (10 min) 4 h (2 h)	Right/5/Throb/NR Left/2/Pres/+ Unilat^7^/Throb/+	−/+/− −/+/− +/+/+	Yes Yes	Yes (20 min) No (NA)	2 x Treo (3 h) / Yes, Sumatriptan 50 mg (4 h) / Yes None
8 EM	100/50/75/75	CGRP Placebo Spon	8 h (5 h) 50 min (10 min)	Left/9/Pres/+ Bilat/2/Pres/M Left/Throb/+	+/+/+ −/+/− +/+/+	Yes No	Yes (2 h) No (NA)	None None
9 CM	75/0/75/75	CGRP Placebo Spon	4 h (5 h) None	Right/2/Throb/+ ᅟ Bilat+Unilat^h^/Throb/+	−/−/− ᅟ +/+/+	Yes	No (NA)^e^	None
10 CM	100/0/25/100	CGRP Placebo Spon	None None	Right/Throb/+	+/+/+			
11 CM	0/0/0/0	CGRP Placebo Spon	50 min (10 min) None	Bilat/5/Throb/M Left/Throb/+	−/−/− +/+/+	No	No (NA)	Panadol Extra + Ibuprofen 600 mg (2 h) / Yes
12 CM	50/25/0/25	CGRP Placebo Spon	6 h (1 h) None	Right/9/Throb/+ Right/Throb/+	+/+/+ +/+/+	Yes	Yes (40 min)	2 x Treo + Paracetamol 1 g + Meto 10 mg (6 h) / Yes
13 CM	50/50/75/50	CGRP Placebo Spon	80 min (20 min) 7 h (1 h)	Right/5/Throb/+ Bilat/2/Pres/+ Right/Throb/+	−/+/+ −/−/− +/+/+	Yes No	Yes (60 min) No (NA)	Sumatriptan 100 mg + Naproxen 500 mg (2 h) / Yes None

Efficacy score: Reduction in migraine days/reduction of the headache intensity/reduction in headache days/reduction in days of used rescue medication. Headache characteristics: Localization/intensity/quality/aggravation. Associated symptoms: Nausea/photophobia/phonophobia. The criteria for a migraine-like attack are described in ‘Methods’. Treatment efficacy: ≥ 50% decrease of headache intensity within 2 h

*Bilat* Bilateral, *Throb* Throbbing, *Pres* Pressing, *M* Missing data, *NR* Not reported, *CM* Chronic migraine, *EM* Episodic migraine

*KP* Codeine 30.6 mg + Paracetamol 500 mg, *Panadol Extra* Paracetamol 500 mg + Caffeine 65 mg, *Treo* Aspirin 500 mg + Caffeine 50 mg, Meto: Metoclopramide 10 mg, Riza: Rizatriptan 10 mg

^a^2–12 h data not reported; ^b^ 3–4 h data not reported; ^c^ 2–12 h data not reported for aggravation and mimics usual migraine; ^d^ Sleep at 8–9 h, headache intensity score was 1 at 10 h; ^e^ Possible migraine-like attack; ^f^ 11–12 h data missing, but reported pain relief and sleep after medication intake; ^g^ Unilateral, no side preference; ^h^ Shifting between bilateral and unilateral (no side preference)

The three patients, who did not develop migraine-like attacks after CGRP, were chronic migraine patients (patients 9, 10 and 11). One of these patients (patient 11) was a poor responder with an efficacy score of zero for all four outcome variables. The other two patients were high responders (patients 9 and 10).

**Fig. 5:**
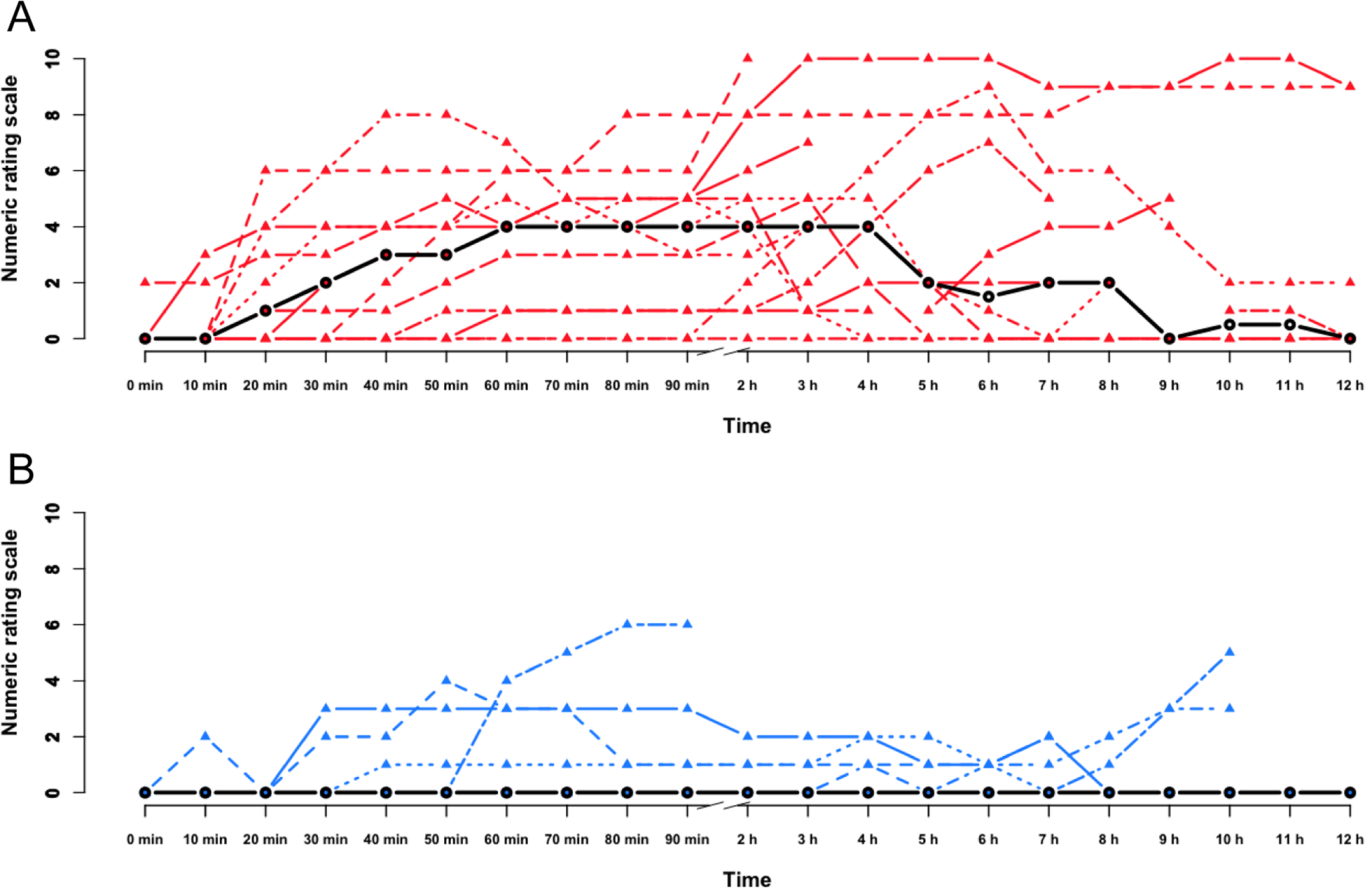
Headache intensity after CGRP and placebo. Individual headache intensity scores on the CGRP day (**a**) and placebo day (**b**). Black lines: Median intensity at each time point. The median headache intensity was 0 for all time points after placebo. The median time (range) to onset of migraine was 50 min (20–152.5) after CGRP

The AUC for headache intensity was greater after CGRP compared to placebo at both 0 to 90 min (*p* = 0.009) and 2 to 12 h (*p* = 0.014) (Fig. 5). The median peak headache intensity score was 5 (5 to 9) after CGRP, compared to 2 (0 to 4) after placebo (*p* = 0.004). Time to peak headache was 180 min (110 to 270) after CGRP and 330 min (72.5 to 660) after placebo (*p* = 0.250).

### Vital signs and adverse events

The AUC for HR was higher (*p* < 0.001) and AUC for MAP was lower (*p* < 0.001) after CGRP compared to placebo. All patients reported warm sensations (13/13 (100%)) after CGRP compared to only one patient reporting warm sensation (1/13, (8%)) after placebo (*p* < 0.001). Flushing was observed after CGRP in all patients (13/13 (100%)) compared to none after placebo (*p* < 0.001). Five of 13 patients (63%) reported palpitations after CGRP, compared to two of 13 (15%) after placebo (*p* = 0.014).

### Predictive values, sensitivity and specificity

Positive predictive value for CGRP-induced attacks in erenumab high responders was 0.80 (95% CI 0.49 to 0.96) and sensitivity was 0.80 (95% CI 0.66 to 0.89). Negative predictive value was 0.33 (95% CI 0.08 to 0.73) and specificity was 0.33 (95% CI 0.01 to 0.91).

## Discussion

Our major finding was that patients with response to erenumab showed hypersensitivity to CGRP infusion in a placebo-controlled experiment. In addition to a high migraine induction rate (77%), compared to previous studies, participants also reported moderate to severe (median peak intensity of 5, range 5 to 9) and long-lasting headaches (Fig. 5), which further points toward high CGRP susceptibility. Previous studies reported median peak headache intensities ranging from 1 to 4. [9, 10]

Mechanisms of migraine initiation by CGRP and migraine prevention by anti-CGRP mAbs are unknown. Calcitonin gene-related peptide is expressed in the trigeminal C fibers [17], trigeminal ganglion [18] and trigeminal nucleus caudalis [19], and its receptors are expressed in vascular smooth muscle cells [20], A-delta fibers [17] and trigeminal ganglia. [18] Calcitonin gene-related peptide binds to its receptor and activates multiple intracellular signaling pathways of which the most well-known is activation of adenylate cyclase and formation of cyclic adenosine monophosphate (cAMP). [21] In arteries, this leads to dilation through an endothelial-dependent synthesis of nitric oxide or relaxation of vascular smooth muscle cells via opening of ATP-sensitive potassium channels (Fig. 1). [22, 23] In trigeminal ganglion cells, the cAMP increase may cause sensitization of nociceptive neurons through upregulation of gene transcription and algogenic receptors in the cell membranes. [21, 24] In healthy volunteers, CGRP modulates inputs from noxious heat stimulation of the trigeminal area in the brain stem and insula. [25] The phosphodiesterase-3 inhibitor, cilostazol, potentiates the accumulation of cAMP in a receptor independent manner, and induces migraine in 86% of patients [26, 27], supporting the notion that cAMP upregulation may induce migraine. To what extent erenumab interacts with these mechanisms and exerts its anti-migraine effect is not fully clarified. Interestingly, erenumab inhibits CGRP-driven increases in dermal blood flow after capsaicin injections suggesting peripheral effects of CGRP receptor blockage. [28]

Our study explored a possible association between self-reported erenumab efficacy and sensitivity to migraine induction by CGRP. Identifying a link between poor response to mAb treatment and not developing migraine when challenged with CGRP (a so-called *non-CGRP phenotype*) could provide a biomarker for treatment response. In an effort to provide test reliability measures, we calculated predictive values, sensitivity and specificity as post hoc analyses. Positive predictive value and sensitivity for CGRP-induced attacks in erenumab high responders were high. In contrast, negative predictive value and specificity were low, impaired by the small sample of erenumab poor responders. We evaluated erenumab treatment response using four variables: reduction in migraine days, reduction in headache intensity, reduction in headache days and reduction in days using rescue medication. Our predefined criteria for “poor response” identified three such participants (subjects 5, 11 and 12 in Table 1). One of these was a *non-responder* who scored zero in all four efficacy variables. This participant reported no migraine after CGRP infusion. The other two poor responders reported migraine-like attacks after CGRP. We obtained treatment efficacy from 19 patients (Fig. 3) and only the three above-mentioned patients reported “poor response”. Therefore, we could not include enough poor responders to calculate a correlation to low migraine induction, which is a limitation. Furthermore, we cannot ignore the fact that having a poor response to erenumab in mAb trials might affect a patient’s willingness to participate in our study, subsequently leading to sampling bias. Our findings suggest that having a positive response to erenumab, based on our questionnaire variables, is associated with a high susceptibility to migraine induction by CGRP. The lack of a larger group of poor responders inhibits us from drawing conclusions on a possible association between those patients and a low susceptibility to CGRP. The question remains whether a CGRP provocation model can be used to predict efficacy of anti-CGRP mAb treatment when it becomes available. A large-scale prospective provocation study in patients, before they receive anti-CGRP treatment, would allow us to draw conclusions on poor responders i.e. patients with a possible *non-CGRP* migraine phenotype. When a sufficient number of *non-responders* have been provoked, we will be able to determine if the CGRP model of migraine is a biomarker for treatment response. Consequently, we will be able to provide biomarker reliability tests with sensitivity and specificity as outcome measures.

## Conclusion

In this study we showed high migraine induction capabilities with CGRP in migraine patients who responded to erenumab treatment compared to data from previous CGRP provocation experiments. [8–11] If an association between poor migraine induction and poor treatment efficacy is also evident, the CGRP model of migraine could become the basis for a biomarker for mAb treatment response. Such a biomarker would be a powerful tool for clinicians choosing therapeutics for the prevention of migraine.

## Abbreviations

AUC: Area under the curve
cAMP: Cyclic adenosine monophosphate
CGRP: Calcitonin gene-related peptide
HR: Heart rate
ICHD-3 beta: International classification of headache disorders version 3 beta
mAb: Monoclonal antibody
MAP: Mean arterial pressure
NRS: Numerical rating scale
